# Fiber-Optic Refractometer Based on an Etched High-*Q* π-Phase-Shifted Fiber-Bragg-Grating

**DOI:** 10.3390/s130708827

**Published:** 2013-07-10

**Authors:** Qi Zhang, Natale J. Ianno, Ming Han

**Affiliations:** Department of Electrical Engineering, University of Nebraska-Lincoln, Lincoln, NE 68588, USA; E-Mails: qzhang@huskers.unl.edu (Q.Z.); nianno1@unl.edu (N.J.I.)

**Keywords:** fiber gratings, optical fiber sensors, optical resonators, refractive index

## Abstract

We present a compact and highly-sensitive fiber-optic refractometer based on a high-*Q* π-phase-shifted fiber-Bragg-grating (πFBG) that is chemically etched to the core of the fiber. Due to the π phase-shift, a strong πFBG forms a high-*Q* optical resonator and the reflection spectrum features an extremely narrow notch that can be used for highly sensitivity refractive index measurement. The etched πFBG demonstrated here has a diameter of ∼9.3 μm and a length of only 7 mm, leading to a refractive index responsivity of 2.9 nm/RIU (RIU: refractive index unit) at an ambient refractive index of 1.318. The reflection spectrum of the etched πFBG features an extremely narrow notch with a linewidth of only 2.1 pm in water centered at ∼1,550 nm, corresponding to a *Q*-factor of 7.4 × 10^5^, which allows for potentially significantly improved sensitivity over refractometers based on regular fiber Bragg gratings.

## 1. Introduction

Refractive index (RI) sensors based on various types of fiber Bragg gratings (FBGs) have been extensively studied in the past decade for their potential applications in biochemical sensing [1–9]. In order to perform RI measurements, the fiber diameter is reduced to expose the evanescent field to the ambient environment. These RI sensors are demodulated based on either wavelength measurements [1,3,4] or light power/intensity measurements [7–9]. For wavelength-based RI grating sensors, the ambient RI change is detected typically by a shift in the Bragg wavelength of the FBG. The detection limit is one of the most important parameters for characterizing a RI sensor. For a wavelength-demodulated FBG-based RI sensor, the detection limit is determined by two factors: (1) the RI responsivity [defined herein as the wavelength shift caused by a RI unit (RIU)] and (2) the measurement resolution of the Bragg wavelength shift. Both factors have been used to improve the RI detection limit. The RI responsivity can be increased by etching the fiber to a smaller diameter to increase the evanescent field in the ambient environment [3]. To improve the spectral measurement resolution, the FBG spectral width needs to be reduced. It has been shown that, for a biochemical sensor based on an optical resonator, the minimum detectable wavelength shift Δλ_min_ is inversely proportion to the quality factor (Q) of the resonator when an intensity–noise–limited wavelength detection scheme was used (*i.e.*, using a wavelength scanning laser and a photodetector) [10]. Unfortunately, the linewidth of a FBG reflection peak is relatively wide (*i.e.*, greater than 100 pm for a 1-cm FBG at 1,550 nm, corresponding to a Q < 1.55 × 10^4^), which limits the spectral measurement resolution. To reduce the linewidth of the spectral feature, FBG sidelopes, which may have a sharper feature than the main FBG reflection peak, have been used in a FBG-based biosensor to improve Δλ_min_[6]. However, the improvement is rather limited. An alternative way is to use two FBGs fabricated on the same fiber to form an in-line fiber Fabry-Perot interferometer (FFPI) and the sharper interferometric fringes have been demonstrated to improve the detection limit [4]. In such a case, only the portion of the fiber between the two FBGs was etched. However, the resonance linewidth of the demonstrated FFPI sensors was still broad (∼25 pm, corresponding to Q ∼ 6.2 × 10^4^) in water, possibly limited by the large optical loss due to the fiber diameter mismatch between the etched fiber and the unetched FBGs. Moreover, as the resonance shifts away from the Bragg wavelength of the FBG, where the reflection is highest, the Q of the resonance is reduced. In addition, the demonstrated FFPI sensors have multiple resonant peaks and increased sensor size, which may cause ambiguity in sensor interrogation and limit their applications as point sensor devices.

**Figure 1.**
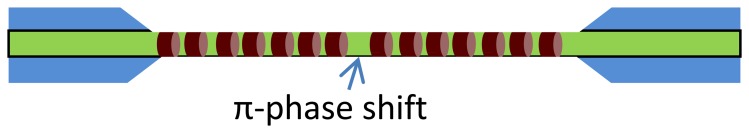
Schematic of a πFBG RI sensor.

Here we propose and demonstrate a fiber-optic RI sensor based on a chemically-etched π-phase-shifted FBG (πFBG) with compact size and a significantly sharper resonant feature compared to other FBG or FFPI sensors. The structure of the πFBG sensor is schematically shown in Figure 1. Different from a regular FBG consisting of periodic RI modification in the fiber core, a πFBG has a π-phase discontinuity in the center of the grating. For strong gratings, the introduction of the phase discontinuity leads to an extremely narrow notch in the reflection spectrum at the Bragg wavelength given by:

(1) $$\lambda_{B}=2n_{\text{eff}}\Lambda$$

where n*_eff_* is the effective refractive index of the optical mode guided by the fiber and Λ is the period of the grating. Different from FFPI sensors, a πFBG has only a single longitudinal resonance mode and a high Q–factor can be obtained with relatively short grating length. The πFBG sensor demonstrated here, centered at ∼1,550 nm and etched to a diameter of ∼9.3 μm, is only 7.6 mm long, and has an approximate linewidth of only 2.1 pm in water, corresponding to a Q = 7.4 × 10^5^, which is more than 10 times larger than the FFPI sensor reported in [4]. We note that a phase-shifted FBG without etching is used as a compact RI sensor in [11], where an otherwise uniform FBG is separated at the center by a gap filled with the liquid being measured. The change of the liquid RI changes the phase shift of the FBG and consequently the wavelength position of the spectral notch. However, the Q-factor of the device studied in this paper is similar to a regular FBG and low, which likely results from the reflection loss on the boundaries between the FBG end-faces and the liquid that form the gap. Moreover, the phase shift changes and typically is not equal to π as the liquid RI changes, which can further reduce the Q-factor as the phase shift is deviated from π.

## 2. Sensor Fabrication

**Figure 2.**
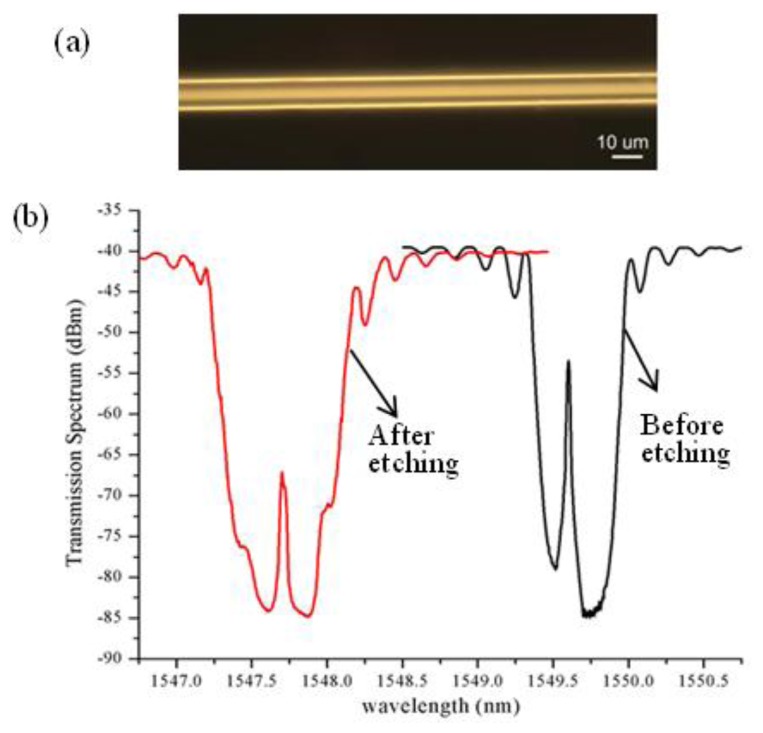
**(a)** Optical microscope image of an etched fiber. (**b**) Transmission spectra of a πFBG measured by an OSA before and after fiber etching.

A small diameter (D = 80 μm) single-mode fiber (SMF) was chosen for the sensor fabrication. Compared to a regular 125 μm fiber, the use of the small diameter fiber can significantly improve the fiber etching efficiency that is performed after the πFBG fabrication. To achieve the πFBG RI sensor structure as shown in Figure 1, a 7.6 mm long πFBG was written on the fiber using a setup and procedure reported earlier [12]. It is based on a scanning 193 nm UV laser beam, an aperture, and a uniform phasemask mounted on a high-precision translation stage. Briefly speaking, the fabrication process consists of three steps. First, a 3.8 mm uniform grating was written by scanning the 193 nm UV-beam which was focused onto the fiber through a cylindrical lens. Then, using the high-precision translation stage, the phase mask was moved by approximately half of the grating pitch, corresponding to the π phase shift of the grating, along the fiber axis direction. The last step was to position the aperture to the other half of grating region and write a uniform grating of the same length by scanning the UV beam again. We note that the use of the 193 nm laser allows us to write high-quality gratings directly on standard germanium-doped fibers without photosensitization through the two-photon UV excitation [13]. After the grating writing, the fiber was etched with a 24% buffered hydrofluoric (BHF) solution. During the etching process, a broadband light source and an optical spectrum analyzer (OSA) were used to monitor the transmission spectrum of the grating. At the beginning, due to the temperature increase from the exothermic reaction of the BHF with SiO_2_, the grating spectrum shifted to longer wavelength. When the fiber was further thinned so that the evanescent field was exposed to the ambient solution, the grating wavelength started to shift rapidly toward shorter wavelength as the waveguide effect on the effective RI of the mode became dominant. Such spectral evolution during the fiber etching was also observed in [14]. Figure 2a is an optical microscope image of the etched fiber, indicating the fiber diameter was approximately 9.3 μm. Figure 2b shows the transmission spectra of the πFBG in air before and after the fiber etching measured by the OSA, showing that the grating wavelength shifted by ∼2 nm toward shorter wavelength after etching. The characteristic transmission peaks at the center of the πFBG spectra are evident. We also noticed that etching process changed the spectral shape; e.g., the spectrum was more symmetric around the center peak after the fiber etching. The depth of the transmission spectra of the etched πFBG is approximately 45 dB, from which a maximum reflectivity of 99.997% is estimated for the πFBG (ignoring the grating loss and the loss due to the mode-field match between the etched and unetched fiber). With such a high grating reflectivity, extremely narrow spectral feature of the πFBG is expected.

**Figure 3.**
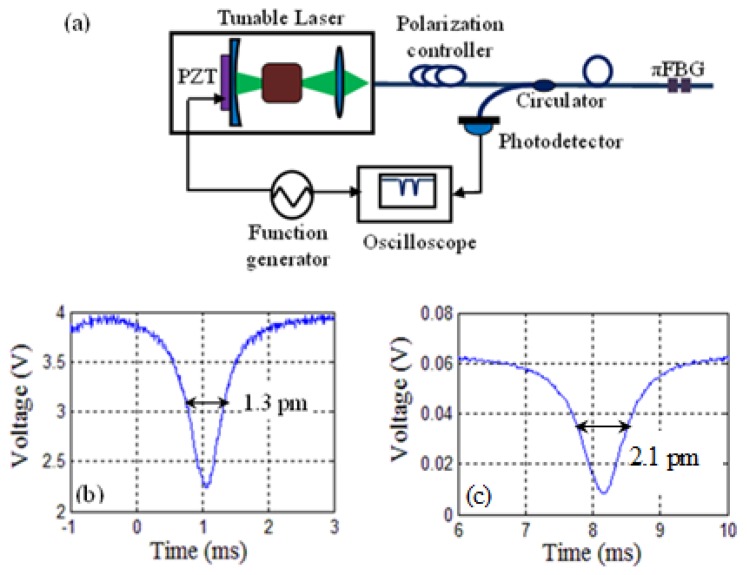
**(a)** Experimental setup to measure the reflection spectral notch of an πFBG sensor. The wavelength scanning speed was 1.6 pm/ms. (**b**) and (**c**) are the spectral notches measured using the setup shown in (a) for an πFBG sensor before and after fiber etching, respectively. (c) was obtained when the sensor was immersed in water.

The linewidth of the πFBG reflection spectral notch cannot be measured accurately by the OSA because of the limited resolution (20 pm) of the OSA. Instead, a narrow-linewidth (<300 kHz) external-cavity tunable laser whose wavelength was tuned through a built-in PZT transducer by a triangle voltage wave, was used to measure the reflection spectrum around the πFBG reflection notch, as schematically shown in Figure 3a. The measurement results before and after fiber etching are shown in Figure 3b and 3c respectively. The linewidth of the πFBG spectral notch before fiber etching was ∼1.3 pm, corresponding to a Q = 1.2 × 10^6^. After fiber etching, the spectral notch was noticeably broadened but still had a linewdith of only 2.1 pm (Q = 7.4 × 10^5^), which was more than 10 times narrower than the spectral feature of the FFPI sensor reported in [4].

There are at least two mechanisms that contribute to the reduced Q-factor of the etched πFBG from the unetched one. The first is the large absorption of water at wavelengths around 1,550 nm. To further increase the Q, πFBGs of shorter Bragg wavelengths, where the absorption of water is smaller, may be used. The second is the surface roughness induced by the BHF etching process. We notice that the spectral width of the πFBG reflection notch rapidly increased as the fiber was further thinned to the germanium-doped fiber core. Rough surface has been shown following wet etching of germanium-doped fiber core of graded-index multimode fibers [15]. It is also shown that smooth fiber core surface could be achieved by optimizing the pH of the BHF solution combined with ultrasonic agitation [15], which can also be applied in processing the πFBG sensor here to improve the surface quality on fibers with further reduced diameter.

## 3. Sensor Test

**Figure 4.**
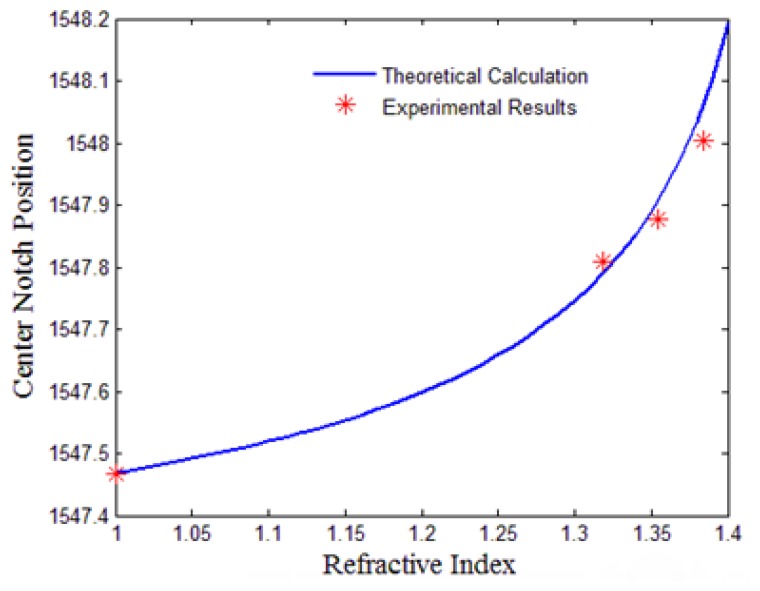
Experimental and simulated spectral notch position as a function of the RI of ambient material.

We firstly tested the sensor response to large RI changes. The πFBG sensor was placed in air and liquids of different RIs (water, ethanol, and 40 w.t.% glycerol solution in water) and we measured the πFBG transmission spectrum using a broadband source and an OSA (the same setup in obtaining Figure 2b. Theoretical analysis was also carried out with the following πFBG parameters: fiber core diameter: 9.78 μm; fiber core RI: 1.451; and grating pitch: 535 nm. More specifically, the surrounding environment was considered as the cladding of the fiber and the effective refractive index of the fundamental mode was calculated against the refractive index of the cladding based on a two4ayer optical fiber model, from which the wavelength position of the πFBG center notch was obtained by Equation (1). The experimental results and the simulated curve for the wavelength of the πFBG transmission peak as a function of the RI to be measured are shown in Figure 4 and they agree with each other well.

**Figure 5.**
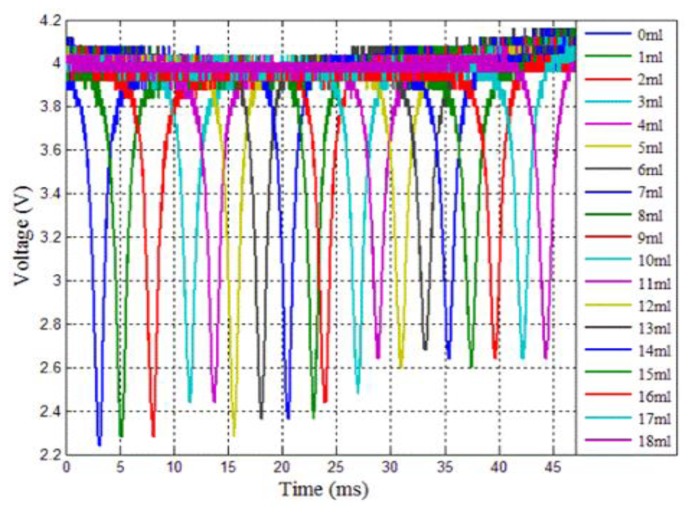

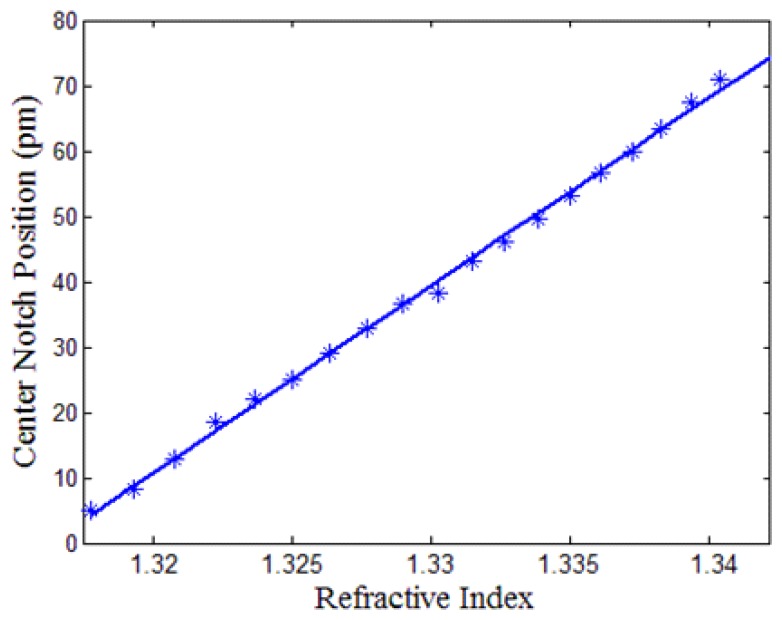
**(a)** Reflection spectra of the πFBG sensor in glycerol-water solutions of different concentrations. Note that a time elapse of 1 ms corresponds to a wavelength shift of 1.6 pm. (**b**) Spectral notch shift as a function of the solution RI and its linear least squares fitting.

To demonstrate the proposed πFBG for high-sensitivity RI measurement due to the extremely sharp spectral feature, sensor response to small RI changes was studied. To achieve this, we placed the sensor in 100 mL of deionized water and subsequently added glycerol into the liquid to change the RI of the solution. Before each spectral measurement, 1 mL glycerol was added to the liquid, which increased the RI of the liquid by 0.0015. Figure 5a shows the spectra measured each time after the RI was changed. The measurement was achieved using the same setup shown in Figure 3a. As expected, the spectral notch shifted to longer wavelengths as the refractive index increased. It is noted that, in Figure 5a, the 0.0015 RI change induced by the 1 mL glycerol shifted the πFBG spectrum by more than the linewidth of the spectral notch. Figure 5b shows the spectral notch wavelength shift as a function of RI change and the linear least squares fitting, from which the sensor responsivity is found to be 2.9 nm/RIU. Although many regular FBG sensors with smaller fiber diameters used in other works have higher responsivity, the πFBG sensor demonstrated here can still achieve excellent performance in terms of RI detection limit due to the much sharper spectral features enabled by the high-*Q* πFBG that leads to significantly improved measurement resolution. For example, assuming the minimum detectable wavelength shift is 1/50 linewidth (0.04 pm), the RI detection limit of the πFBG sensor used here is 1.4 × 10^−5^.

It is worth noting that variation of the ambient temperature is another limiting factor to the RI detection accuracy and limit. Assuming that the πFBG has a temperature responsivity of ∼1 pm per °C [8], the temperature variation needs to be smaller than 0.0036 °C in order to achieve a RI detection limit of 1.4 × 10^−5^(corresponding to 0.04 pm wavelength shift). Such temperature capability can be readily achieved using commercial precision temperature controllers.

## 4. Conclusions

We have proposed and demonstrated a compact and high-sensitivity fiber-optic RI sensor based on a wet-etched high-*Q* πFBG. The reflection spectrum of the πFBG has an extremely narrow notch whose wavelength is dependent on the RI of the ambient media. The narrow linewidth of the notch significantly increases the measurement resolution of the wavelength shift, contributing to improved detection limit. The πFBG sensor demonstrated here is 7.6 mm long and has a linewidth of only 2.1 pm, corresponding to a *Q* of 7.4 × 10^5^, which is at least 10 times larger than previously reported sensors based on regular FBGs. The proposed RI sensors may find applications in many fields such as biomedical research, environmental monitoring, and industrial process control.
